# Knowledge and attitudes about rabies in dog-bite victims in Bangladesh

**DOI:** 10.1016/j.onehlt.2020.100126

**Published:** 2020-03-09

**Authors:** Ahmed Nawsher Alam, Mahmuda Siddiqua, Jordi Casal

**Affiliations:** a Institute of Public Health, Mohakhali, Dhaka 1212, Bangladesh; b Ibn Sina Medical College, Kallyanpur, Dhaka 1215, Bangladesh; c Departament de Sanitat i Anatomia Animals, Facultat de Veterinària, Universitat Autònoma de Barcelona, Bellaterra, Barcelona, Spain; d IRTA, Centre de Recerca en Sanitat Animal (CReSA, IRTA-UAB), Bellaterra, Spain

**Keywords:** Rabies, Bangladesh, Dog bites, Attitudes, Post-exposure prophylaxis

## Abstract

Rabies is an important zoonotic disease that causes several thousand deaths in Asian countries. Bangladesh launched an elimination programme in 2010 based on the mass vaccination of dogs, management of dog bites, application of post-exposure prophylaxis and communication and social mobilization. The aim of this study is to ascertain the behaviour of and knowledge about dog-bite victims.

A cross-sectional descriptive study was performed on 885 dog-bite victims who presented themselves for post-exposure rabies vaccination to six randomly selected vaccination centers, in addition to a tertiary-level hospital in Bangladesh. Most dog-bite victims were male (70%) and with very low or no education qualifications (75%). Respondents' knowledge of rabies was low: 58% were unaware of the consequences of a dog bite and 52% did not know about any indication of rabies. Most knew that rabies in humans can be prevented after dog bites, but up to 70% did not give a correct answer for other questions related to the prevention and treatment of rabies. Knowledge and attitudes about rabies is closely related to level of education. Finally, 58 of those surveyed (6.4%) did not complete the post-exposure prophylaxis correctly.

In conclusion, knowledge about rabies among Bangladeshi citizens is low. An intensive plan to inform and educate people about dog bites, the risk of rabies and measures to adopt for preventing the disease should be implemented in order to reduce risk, including the need to complete post-exposure treatment.

## Highlights

• Knowledge about rabies in victims of dog bites in Bangladesh was low (58% were unaware of the consequences of a dog bite).
• Up to 70% of bite victims did not give a correct answer to some questions related to prevention and treatment of rabies.
• A small but significant proportion of dog-bite victims (6.4%) did not complete the Post-Exposure prophylaxis correctly.

## 1 Introduction

Rabies is a zoonotic disease caused by a virus belonging to *Rhabdoviridae* family and *Lyssavirus* Genus. Humans become infected after a bite or scratch from a rabid animal, usually a dog [1,2]. Rabies is almost always fatal once symptoms have started, but can easily be prevented with pre- and post-exposure prophylaxis. According to the World Health Organization [3], more than 15 million people worldwide receive a post-exposure prophylaxis (PEP) annually. Despite the vast number of treatments, rabies is still the cause of about 59,000 deaths annually (95% Confidence Intervals: 25,000-159,000), 60% of these in Asia [4]. In 2015, the WHO, the Food and Agriculture Organization (FAO), the International Organization for Animal Health (OIE) and the Global Alliance for Rabies Control (GARC) launched a program to achieve “zero human deaths from dog-transmitted rabies by 2030” [5].

Prior to this program, certain countries in South-East Asia had begun rabies-elimination campaigns. Bangladesh launched an elimination program in 2010 with the target of regional elimination by 2020. Bangladesh is located in the Indian subcontinent, a hotspot for rabies, with an estimated canine population of 1.6 million (83% considered to be free-roaming dogs). Annual incidence of humans bitten by dogs is greater than 300,000 [6]. Before the start of the program, more than 2000 people died annually from rabies. In 3 years, human death from rabies decreased by 50%, with the number of reported rabies-related deaths being 52 in 2016.

The program is based on the mass vaccination of dogs, management of dog bites, increased availability of PEP, and communication and social mobilization [7]. This last point includes promoting awareness and increasing people's knowledge about the transmission and prevention of rabies. Knowing the circumstances in which dog bites have occurred, as well as a clear understanding of people's level of knowledge and of their beliefs is important for planning communication strategies aimed at increasing the awareness of rabies and what measures can be taken against it, particularly in rural areas [8].

As in other countries, dog bites are common in Bangladesh; however, there are misconceptions and false beliefs about such incidents among the general population, and there is also a lack of awareness about rabies. The aim of this study is to characterize dog-bite victims, to know ascertain their behavior and knowledge and to determine the factors associated with a failure to complete full PEP.

## 2 Materials and methods

### 2.1 Study area

**Fig. 1:**
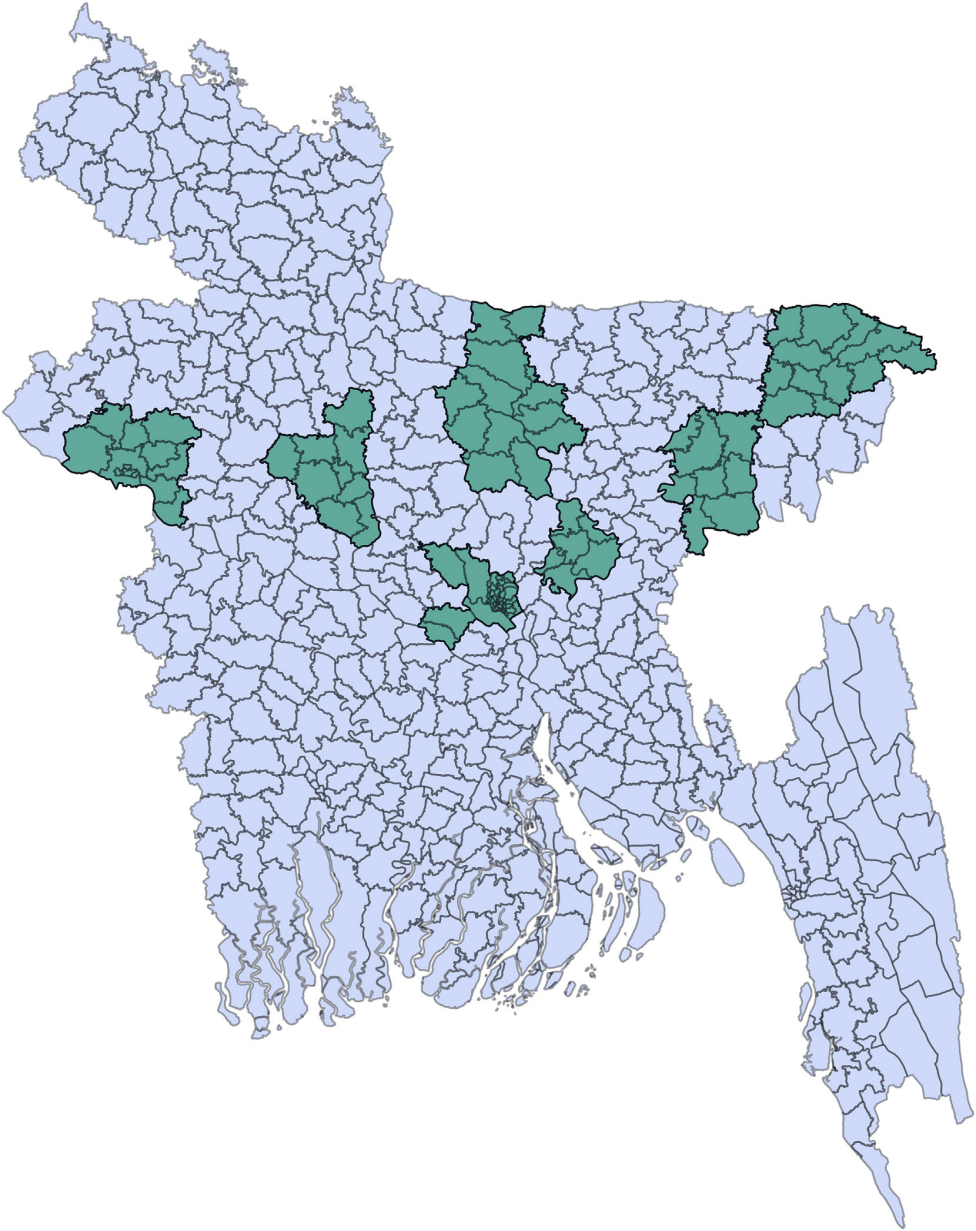
Geographic distribution of the seven districts in which data were collected.

Bangladesh is divided into 8 administrative divisions and 64 districts. For this study, three divisions (Dhaka, Sylhet, and Rajshahi) were randomly selected, with two districts from each division (Narshingdi, Mymenshing, Sylhet, Habigonj, Sirajgonj and Rajshahi) then also being randomly selected. Finally, a tertiary-level hospital (the Infectious Disease Hospital, Dhaka city) was also included as a referral hospital, making a total of 7 sites (Fig. 1).

### 2.2 Data collection and analysis

A cross-sectional descriptive study was performed based on everyone that went for an initial dose of rabies post-exposure vaccination to the seven selected vaccination centers between 31 March 2013 and 11 July 2013. All patients residing in the study areas were asked to complete a questionnaire; those that consented to be interviewed were then eligible, with a total of 885 cases finally being included in the study. A structured questionnaire with a small number of open questions was used. Interviewers were hired in each center and were trained before start the study by the project team with respect to dog bites and rabies in people and animals. For children aged under 15, the corresponding parents or guardians were interviewed.

Each individual was followed up until the whole PEP protocol was completed. Those who did not attend the vaccination center for entire treatment protocol were interviewed by telephone or, if necessary, visited at home to obtain information on the discontinuation of vaccination. The questionnaire was pre-tested by the project team on subjects who had attended a vaccination center for PEP before the start of the study.

Data were entered into an Epi Info database (Epi Info version −7) and descriptive analyses (proportions and chi-square test) were performed with this software. Multivariable analysis to evaluate the effect of the different variables on knowledge and attitudes was conducted with Stata. Briefly, a bivariate chi-square test was performed between these variables and the explanatory variables. All variables with a *p*-value <.25 were selected and tested to detect co-linearity. For variables that presented strong co-linearity (*p* < .05), only the variable with the stronger initial association was retained and included in the logistic regression analysis. Using a backward selection, all variables with a nonsignificant *p*-value biological association were sequentially deleted from the model.

## 3 Results

### 3.1 Demographic characteristics of dog-bite victims

This study included 885 cases of dog-bite victims who attended a PEP vaccination center in the seven study areas. Table 1 shows the main characteristics of the affected subjects. Males (617 out of 885, 70%) were more common in the study population than females. Ages ranged from 1 year to 95 years with a median age of 20 years. Due to the high proportion of young people (386–43.6%- were younger than 18 years); the number of students is high (284, 32%). This was the most frequent occupation of the patients, followed by housewives (139, 16%) and pre-school children (101, 11%). Most dog-bite victims had very few or no education qualification (662, 75%), and only 41 (8%) finished secondary education or had a higher-education degree.

**Table 1:** Characterization of the 885 dog-bite victims attended to in the PEP vaccination centres.

Variable	Categories	All population	18 years or older
Gender	Males	617 (70%)	330 (66%)
	Females	268 (30%)	169 (34%)
Age	<10	218 (25%)	–
	10–20	200 (23%)	37 (7%)
	20–29	153 (17%)	153 (31%)
	30–39	104 (12%)	104 (21%)
	40–49	88 (10%)	88 (18%)
	50–59	76 (9%)	76 (15%)
	> 60	46 (5%)	46 (9%)
Occupation	Student	284 (32%)	32 (6%)
	House wife	139 (16%)	120 (24%)
	Pre school	101 (11%)	–
	Farmer	88 (10%)	84 (17%)
	Business	70 (8%)	70 (14%)
	Service	57 (6%)	54 (11%)
	Employee	30 (3%)	28 (6%)
	Beggar/ Do nothing	26 (3%)	15 (3%)
	Other	76 (9%)	83 (17%)
	Unknown	14 (2%)	13 (3%)
Education	No Education	267 (30%)	150 (30%)
	Primary school	395 (45%)	176 (35%)
	Secondary school	145 (16%)	101 (20%)
	High school	43 (5%)	41 (8%)
	Graduate	28 (3%)	28 (6%)
	Other	7 (1%)	3 (1%)
Type of house	House /apartment	163 (18%)	92 (18%)
	Tin shed	661 (75%)	371 (74%)
	Hut	55 (6%)	32 (6%)
	Other	6 (1%)	4 (1%)
Location	Rural	545 (62%)	302 (61%)
	City	327 (37%)	190 (38%)
	Slumps	13 (1%)	7 (1%)

### 3.2 Epidemiology of dog bites

All victims were bitten by dogs, usually free-roaming animals such as community dogs (245, 27.7%) or stray dogs (291, 32.9%). Two hundred fifty-three (29%) were bitten by their own dog (72 people) or by the neighbors' dog (181), and 96 (10.8%) did not know the ownership of the animal. The highest proportion of people were bitten outside their home, most of them (440, 50%) in the street (Table 2).

**Table 2:** Characterization of circumstances of bite in 885 dog-bite victims.

		Number	Proportion
Ownership of the dogs	Own (pet Dog)	72	8.1%
	Neighbour's Dog	181	20.5%
	Community Dog	245	27.7%
	Stray Dog	291	32.9%
	Unknown	96	10.8%
Place	At street	440	49.7%
	At home	361	40.8%
	At market	48	5.4%
	At school	13	1.5%
	Others	23	2.6%
Activity when bitten	Walking	629	71.1%
	Stepping over	95	10.7%
	Playing with	37	4.2%
	Feeding	22	2.5%
	Provocating	9	1.0%
	Others	93	10.5%
Anatomic region	Leg	705	79.7%
	Hand	114	12.9%
	Body / neck	74	8.4%
	Head	6	0.7%

Most dog bites occurred without provocation (629, 71.1%); however, some cases were caused by accidentally stepping on a dog (95, 10.7%) or by children irritating a dog (9 cases). Very few cases happened when either feeding dogs or playing with them. Almost 80% of the bites (705 cases) were on the leg (Table 2), but this aspect shows differences between ages: 65% of children under 10 years were bitten on the leg, while the proportion was 85% for older people (chi squared = 40.1, *p* < .0001).

Subjects were also asked about previous cases of dog bites in the family: 77 respondents (8.7%) informed that a relative had been bitten by a dog within the last 12 months, mainly by non-pet dogs (58), neighbors' dogs in 12 cases, with only 7 bitten by their own dog. Of these bite victims, only 28 undertook treatment after the bite; 29 undetook no treatment and the remaining 20 did not know whether treatment had been undertaken.

### 3.3 Knowledge and attitudes about rabies

Five hundred sixteen (58%) of the subjects interviewed were unaware of the consequences of a dog bite. Only 219 (25%) were able to name the disease (176 termed it “hydrophobia”, 43 “rabies”); other responses were less specific, indicating that bites can lead to madness (48), death (44) or disease (23). According to a local belief, 35 respondents answered that they could become pregnant, and in some cases that such a pregnancy will produce a puppy (13 respondents). Regarding their knowledge about the clinical signs of rabies in dogs, 460 (52%) did not know any signs, 262 (30%) mentioned salivation, 163 (18%) indicated aggressive behavior and 106 (12%) referred to restlessness (Table 3).

**Table 3:** Knowledge about rabies in the surveyed dog-bite victims.

	Knowledge about rabies	Number	Proportion
Name of the disease	Hydrophobia	176	19.9%
	Rabies	43	4.9%
	Death	44	5.0%
	Become mad	48	5.4%
	Become pregnant	35	4.0%
	pregnant with a puppy	13	
	Disease	23	2.6%
	Do not know	516	58.3%
Clinical signs (dogs)	Salivation	262	29.6%
	Aggressive	163	18.4%
	Restless	106	12.0%
	Paralysed	31	3.5%
	Do not take food	21	2.4%
	Sleepy	10	1.1%
	Others	13	1.5%
	Mad	7	0.8%
	Do not know	460	52.0%

When asked directly about rabies, respondents showed slightly better knowledge about the disease, with 467 (53%) knowing that the disease could be transmitted from dogs to humans. Most respondents knew that rabies in humans can be prevented after a dog bite, but up to 70% gave an incorrect answer to other questions regarding the prevention and treatment of rabies (Table 4).

**Table 4:** Knowledge about rabies transmission, prevention and evolution of the disease in humans and dogs in the dog-bite victims.

Questions related to knowledge about rabies	Yes	No	Don't know
Rabies can be transmitted from dogs to humans	467 (53%)	418 (47%)	
Rabies in dogs can be prevented	117 (13%)	90 (10%)	678 (77%)
Rabies in dogs can be cure after they become sick	17 (2%)	238 (27%)	630 (71%)
Rabies in humans can be prevented before dog bite	179 (20%)	89 (10%)	617 (70%)
Rabies in humans can be prevented after dog bite	569 (64%)	65 (7%)	251 (28%)
Rabies in humans can be cured after they get sick	72 (8%)	231 (26%)	575 (66%)

### 3.4 Measures taken after a dog bite

Most of the victims took no measures to treat the bite wound before coming to hospital for vaccination (279, 31.5%). 198 (22.4%) applied antiseptic; 192 (21.7%) applied plain water and 125 (14.1%) applied soap and water. Application of indigenous products such as lime, soda, salt and kerosene oil was also carried out by 129 patients (14.6%).

In 601 cases (67.9%), no action was taken against the dog; in 214 cases (24.2%) the animals could not be traced and in 42 cases (4.8%) the dogs were killed. Only 19 dogs (2.2%) were observed in order to check for rabies. Most of the subjects surveyed (681, 77.0%) did not know the vaccination status of the dog that had bitten them; 193 (21.8%) said that the dogs were not vaccinated and only 10 (1.1%) said that the dogs in question were vaccinated.

### 3.5 Level of education: effect

Level of education had an important effect on knowledge of and attitudes about rabies. A clear gradient can be observed, running from people without education through to graduates having a knowledge of the name and clinical signs of the disease, and those who cleaned the wound with soap and water (Table 5).

**Table 5:** Effect of education on knowledge of and attitudes regarding rabies.

Dependent variable	Independent variables	Beta	SE Beta	Odds ratio	95% IC	Significance
Know the name of the disease	No Education			1		
	Primary	0.0352	0.2166	1.036	0.678–1.584	0.8709
	SSC	1.3016	0.2384	3.675	2.303–5.864	>0.0001
	HSc	2.1032	0.3543	8.193	4.091–16.407	>0.0001
	Graduate	3.7984	0.6336	44.631	12.891–154.523	>0.0001
	Constant	−1.6784	0.1681			>0.0001
Know the clinical signs	No Education			1		
	Primary	0.3408	0.222	1.406	0.910–2.173	0.1247
	SSC	0.3841	0.2786	1.468	0.850–2.535	0.168
	HSc	0.665	0.403	1.944	0.883–4.284	0.0989
	Graduate	1.5712	0.4218	4.813	2.105–11.00	0.0002
	Constant	−1.8589	0.1792			>0.0001
Clean with soap	No Education			1		
	Primary	0.1082	0.2584	1.114	0.672–1.849	0.6754
	SSC	0.8826	0.2868	2.417	1.378–4.240	0.0021
	HSc	0.8557	0.4263	2.353	1.020–5.426	0.0447
	Graduate	2.0428	0.4299	7.712	3.321–17.909	>0.0001
	Constant	−2.1848	0.203			>0.0001

Significances lower than .05 are underlined

### 3.6 Subjects who did not complete PEP correctly

Fifty-eight people did not complete PEP correctly (6.4% of all treatments: 10 people took only 1 dose, 23 took 2 and 20 took 3 but did not complete the entire treatment. The other 5 people completed the 4 doses, but they did not follow the schedule. Of these 58 people, 25 thought they did not need the full protocol for protection; 2 changed to “another treatment”; 7 were too busy; 6 said that they could not afford the treatment; 4 left the city; 3 said that their wound was not a real bite; and 7 could not be traced. Failure to complete the full vaccination protocol could not be related to level of education or any of the other studied variables.

## 4 Discussion

Dog-bite injuries are an important health problem worldwide. In countries where rabies is endemic, in addition to the importance of the bites themselves, they become still more important because they are the principal route of transmission of this disease.

From 2013, between March and July, 883 people were surveyed when they attended seven vaccination centres in Bangladesh to receive the initial dose of rabies PEP. A high proportion of the patients (70%) were male. This is much higher than subjects described in Bhutan [9], but is similar to the 72% obtained in two Indian studies [10,11] and slightly lower than in other Asian countries, such as the 78% and 79% observed in Iran [12] and Pakistan [13], respectively.

Most of the victims were children or young people under 20. These findings are highly similar to those described in several studies conducted both in developed [14,15] and developing [16,17] countries, including previous studies from Bangladesh [18,19]. Increased dog-bite incidence in children is considered a behavioural risk because of children's curiosity, lack of understanding as regards dog behaviour and their inability to protect themselves from attack [20].

Almost three quarters (71.6%) of the dog bites occurred without any previous provocation or interaction by the victim, as has been previously reported [20,21]. In most cases, victims were attacked by free-roaming dogs (71.3%). This is a consequence of the high number of stray dogs in Bangladesh, as commonly observed in developing countries [22,23].

As previously mentioned, dog bites represent a considerable problem in rabies-endemic countries. Rabies is a neglected disease responsible for several thousands deaths annually, and has an important cost deriving from the 15 million people who receive a post-exposure prophylaxis [3], particularly in developing countries. In 2015, the Global Alliance for Rabies Control was launched to change this situation, with the objective of eradicating rabies transmitted by terrestrial animals by 2030. One of the bases of Global Alliance program is the implementation of rabies-awareness campaigns adapted to the local situation of the countries in question, in order to motivate dog owners to vaccinate against rabies, to prevent dog bites and to administer first aid for bite victims, including wound washing and post-exposure rabies injections [3].

This program faces considerable challenges in countries where people's level of knowledge about rabies and the role of dog bites in the virus transmission is low, as is the case of the subjects surveyed here: 58% of the patients interviewed in this study did not know the name of this disease, and only 25% could provide the term hydrophobia or rabies. As regards the clinical signs of rabies in dogs, the most cited symptoms were salivation, and aggressive or restless behaviour; half of the subjects in the study were unable to name any symptom of rabies.

These results are lower than two other studies carried out in Bangladesh, where 64% and 73% of the people had heard about rabies [24,25]. This difference may be due to a distinct formulation of the question, we can assume that most subjects probably know about rabies, but do not relate it directly to dog bites).

The question on the name of the disease was responded to by 35 people as “becoming pregnant”. Of these, 13 further specified “pregnant with a puppy”. This reflects a strong cultural belief in rural areas of Bengal, that people bitten by a dog may develop an asexual puppy pregnancy, and this includes males [26]. This is a further example of the importance of education in reducing false beliefs about rabies.

In contrast to other studies, only 52.8% of dog-bite victims knew that rabies could be transmitted from dogs to humans, a proportion that is much lower than the 90% to 99% described for Sri Lanka, Pakistan, India and Bhutan [9,[27], [28], [29]]. Knowledge about prevention of rabies in dogs as well as in humans was also low in the subjects included in this study. Between 66% and 77% answered “don't know” to these questions. The only exception to this was that 64% of dog-bite victims knew that rabies in humans can be prevented after a dog bite. Nevertheless, 7% answered indicated that, in their opinion, that this was not the case, despite going to the centre for treatment.

A strong relationship was observed between knowledge about rabies prevention and level of education; similar conclusions were obtained by distinct authors [9,24,28]. This knowledge was also related to an urban population and to people living in houses or apartments; but these variables are strongly correlated with the level of education and, in our opinion, the true cause of lack of knowledge is actually education.

All these findings indicate that both general education and knowledge about rabies was low among dog-bite victims, a fact that could hamper the national rabies-control program.

As children are an important proportion of the population who have been bitten, it is important to include education activities within the school curriculum in order to provide information about dog behaviour and wound management, so as to reduce the risk of rabies in the young population [11,16]. At the same time, practical information on rabies prevention and control should be particularly addressed to people with a low economic and cultural level. Additionally, health authorities and hospital personnel should be aware of the important role they play in educating about rabies prevention and control, and most especially the influence they have in ensuring the completion of PEP treatment.

Another important constraint for rabies control is the high proportion of people bitten by free-roaming dogs, as well as the low number of vaccinated dogs. A reduction in stray dogs and a significant reinforcement of vaccination would reduce rabies transmission among dogs and, consequently, lower the risk for people.

Cleaning the dog-bite wound with soap and water is an important measure and is recommended by the WHO. It is an easy response that can be carried out immediately at home in order to remove the rabies virus from the wound and to reduce the chance of being infected by rabies from a rabid dog [3]. In this study, we found that only 14% of dog-bite victims used soap and water to wash the wound, which is much lower than studies carried out in India (31.1%) and Bhutan (45%) [10,30].

A small but significant proportion of the subjects surveyed (6.4%) did not follow the protocol correctly. At all events, this proportion is far lower than the 17% observed in Bhutan [9] or the 40% and 23% described in India [31] for intramuscular and intradermal rabies vaccination, respectively. The most cited explanation for non-completion of treatment was the belief that the full vaccination course was not needed for protection. Other reasons given for non-completion indicate a lack of knowledge about rabies and about the risk implied by such non-completion. Other authors [9,31] have also cited the difficulties expressed by patients with regard to the vaccination itself (loss of wages, forgotten appointments, interference with work obligations, etc.) and the lack of information from clinicians about the need for treatment follow-up. Patients' motivation through better communication and information is therefore crucial in increasing their completion of the full course of vaccination.

A One Health approach should be applied to overcoming the lack of knowledge about rabies shown by Bangladeshi citizens. In addition to reinforcing the application of vaccines and PEP, public awareness should also be increased. it is important to develop an intensive and coordinated plan comprising governmental departments and agencies that involves health, education and livestock, non-government organizations, international institutions and academia to inform and educate people about dog bites, about the risk of becoming infected from the rabies virus and most especially about the measures to adopt in order to reduce risk, including the need to complete PEP treatment [32].

There are certain limitations to our study; the most important is the representativeness of the population attending the district vaccination centre. We assume that they are different from the general population and particularly from people bitten by dogs who did not attend a vaccination centre. We additionally assume that the knowledge and attitudes in this latter group, and perhaps also in the general population, is lower than or—at the most—equal to the subjects in our study.

Two other circumstance may have an influence on responses, the first being the low level of education of most of participants. This plausibly plays a role in certain participants' lack of understanding particular questions and in providing the response “don't know” (which may be true for them but which is also a ‘safe’ option). A second circumstance is that, on some occasions, patient numbers were high and interviewers consequently had less time to interview each subject.

In conclusion, Bangaldeshi citizens' knowledge about rabies is low and it is important to develop an intensive plan to increase people's understanding of dog bites, of the risk of becoming infected from the rabies virus and of the measures to adopt in order to reduce risk, including the need to complete PEP treatment.

## Founding sources

Massey University, the World Bank and the European Union through the Regional One Health Epidemiology Training Program in South Asia, and the Bangladesh Ministry of Health and Family Welfare.

## Policy and ethics

Written and verbal consent was obtained from dog-bite victims or, in the event of a victim being under 15, from their parents or guardian.

As this study involved no intervention beyond providing responses to an interviewer, participants were not exposed to any study-related risk.

## Declaration of Competing Interest

None.
